# Relative Contribution of Different Mitochondrial Oxidative Phosphorylation Components to the Retinal Pigment Epithelium Barrier Function: Implications for RPE-Related Retinal Diseases

**DOI:** 10.3390/ijms22158130

**Published:** 2021-07-29

**Authors:** Michael H. Guerra, Thangal Yumnamcha, Lalit P. Singh, Ahmed S. Ibrahim

**Affiliations:** 1 Department of Ophthalmology, Visual and Anatomical Sciences, School of Medicine, Wayne State University, 540 East Canfield, Detroit, MI 48201, USA; michael.guerra2@med.wayne.edu (M.H.G.); gl5948@wayne.edu (T.Y.); plsingh@med.wayne.edu (L.P.S.); 2 Department of Pharmacology, School of Medicine, Wayne State University, 540 East Canfield, Detroit, MI 48201, USA; 3 Department of Biochemistry, Faculty of Pharmacy, Mansoura University, Mansoura 35516, Egypt

**Keywords:** ECIS, ARPE-19, AMD, DR, RPE, mitochondria, oxidative phosphorylation, uncouplers

## Abstract

Disruption of retinal pigment epithelial (RPE) barrier integrity is involved in the pathology of several blinding retinal diseases including age-related macular degeneration (AMD) and diabetic retinopathy (DR), but the underlying causes and pathophysiology are not completely well-defined. Mitochondria dysfunction has often been considered as a potential candidate implicated in such a process. In this study, we aimed to dissect the role of different mitochondrial components; specifically, those of oxidative phosphorylation (OxPhos), in maintaining the barrier functionality of RPE. Electric cell-substrate impedance sensing (ECIS) technology was used to collect multi-frequency electrical impedance data to assess in real-time the barrier formation of the RPE cells. For this purpose, the human retinal pigment epithelial cell line—ARPE-19—was used and treated with varying concentrations of specific mitochondrial inhibitors that target different steps in OxPhos: Rotenone for complex I (the largest protein complex in the electron transport chain (ETC)); oligomycin for ATP synthase; and carbonyl cyanide-p-trifluoromethoxyphenyl hydrazone (FCCP) for uncoupling ATP synthesis from the accompanying ETC. Furthermore, data were modeled using the ECIS-Zθ software to investigate in depth the effects of these inhibitors on three separate barrier parameters: cell–cell interactions (R_b_), cell–matrix interactions (α), and the cell membrane capacitance (C_m_). The viability of ARPE-19 cells was determined by lactate dehydrogenase (LDH) Cytotoxicity Assay. The ECIS program’s modeling demonstrated that FCCP and thus OxPhos uncoupling disrupt the barrier function in the ARPE-19 cells across all three components of the total resistance (Rb, α, and C_m_) in a dose-dependent manner. On the other hand, oligomycin and thus ATP synthase inhibition mostly affects the ARPE-19 cells’ attachment to their substrate evident by a significant decrease in α resistance in a dose-dependent manner, both at the end and throughout the duration of the experiment. On the contrary, rotenone and complex I inhibition mostly affect the ARPE-19 paracellular resistance R_b_ in a dose-dependent manner compared to basolateral resistance α or C_m_. Our results clearly demonstrate differential roles for different mitochondrial components in maintaining RPE cell functionality in which uncoupling of OxPhos is a major contributing factor to the disruption barrier function. Such differences can be used in investigating gene expression as well as for screening of selective agents that improve the OxPhos coupling efficiency to be used in the therapeutic approach for treating RPE-related retinal diseases.

## 1. Introduction

The retina is a complex and highly regulated sensory tissue, as visual function is integral to the life and survival of sight-dependent organisms like humans. The retina itself is composed of several layers of neural cells, including photoreceptors, bipolar cells, amacrine cells, and retinal ganglion cells [1]. Just beyond the photoreceptors in the retina are the retinal pigment epithelial (RPE) cells—a cell layer as critical to visual functioning as the photoreceptors themselves. The RPE has many important roles in both visual function and health maintenance of the retina, including phagocytic recycling of photoreceptor tips and the absorbance of all light that passes through the retina [2,3]. Furthermore, RPE also participates in the coordination of innate and adaptive immune defense, regulation of glycolytic metabolism of the photoreceptors, and maintenance of a tightly regulated outer blood-retinal barrier between the neuroretina and the systemic blood supply reaching the retina via the choriocapillaris [2,3,4,5]. Controlling the flow of nutrients such as glucose and amino acids from the choriocapillaris to the retina is important for maintaining strict control of the metabolic pathways within the retina [6]. The RPE also acts to restrict the entrance of systemic toxic metabolites to the retina and to remove harmful metabolites produced in the retina [7,8]. 

Damage to the RPE through insults such as aging, oxidative stress, ischemia, and hyperglycemia results in disruption of this outer blood-retinal barrier, which allows for further damage to occur to the weakened RPE and the underlying neuroretina [9,10,11]. RPE dysfunction has devastating effects on the neuroretina and the progression of several retinal disorders such as diabetic macular edema (DME) and age-related macular degeneration (AMD). In DME, a component of RPE barrier loss is related to dysregulated active transport early in the disease process and then to RPE tight junction loss, later, allowing an accumulation of retinal interstitial fluids [12]. In the advanced form of AMD called geographic atrophy, there is atrophy of the RPE in the macular area and thus loss of the vital RPE cells including barrier integrity, causing photoreceptor damage [13]. As understanding of the immunology of AMD has progressed, new therapies for dry AMD and geographic atrophy are being developed to target damaging immune processes, especially the complement cascade [13,14]. On the other hand, RPE barrier dysfunction with tight junction breakdown in wet AMD facilitates choroidal endothelial cells migration to the retina, contributing to retinal neovascularization [13,15]. Despite this great damage known to occur in these retinal degenerative diseases, the pathogenic mechanisms of this dysfunction remain incompletely understood, and only a few therapies for RPE dysfunction are available [13,16,17]. Several molecular mechanisms have been proposed to be involved in the pathogenesis of RPE dysfunction, and mitochondria have often been considered as potential candidates implicated in such a process [18,19]. 

The mitochondrion is the key player in bioenergetics. Mitochondria contain two membranes, the outer and inner membranes, and generate about 90% of the energy required for cellular activities via a process called oxidative phosphorylation (OxPhos). This process occurs in the inner mitochondrial membrane (IMM), wherein five protein complexes (complex I–V) are embedded. Complexes I or II, III, and IV make up the electron transport chain (ETC) by which electrons pass from the electron donor (NADH or FADH2) to the terminal electron acceptor molecular oxygen (O_2_). As the electrons flow, protons are pumped out across the IMM from the mitochondrial matrix to the intermembrane space through complexes I, III, and IV, creating an electrochemical gradient. According to the chemiosmotic theory proposed by the Nobel laureate Peter Mitchell [20], this electrochemical gradient is coupled to the synthesis of ATP via reentry of protons to the matrix through the ATP synthase (complex V). Therefore, any proton leak that is not coupled with ATP synthesis would result in the uncoupling of the ETC from ATP synthesis and dissipation of the energy in the form of heat (thermogenesis). A well-known example of such an uncoupler is trifluoromethoxy carbonylcyanide phenylhydrazone (FCCP), which collapses the chemiosmotic gradient by carrying intermembrane space protons back across the IMM and thus uncouples ETC and ATP synthase [21,22]. 

The ATP synthase and complex I of ETC are the largest protein complexes present in the IMM, consisting of 29 and 45 subunits, respectively [23,24]. The ATP synthase has two functional domains (F_0_ and F_1_). The F_0_ domain is located in the IMM and acts as a proton-driven turbine, while the F_1_ domain is located in the mitochondrial matrix and has the catalytic side for generating ATP. These two parts are connected by two stalks, one is static and peripheral, while the other is central and rotatory to transmit the spin from the F_0_ subunit to the catalytic F_1_ head [25]. The upper part of the peripheral stalk has an oligomycin sensitivity conferral protein (OSCP), which along with the central stalk, ensures the structural coupling between F_0_ and F_1_. This coupling is affected by the antibiotic oligomycin, which prevents both ATP synthesis and hydrolysis [23]. 

On the other hand, inhibition of complex I (NADH:ubiquinone reductase), the main site for electron insertion into the mitochondrial ETC, is accompanied by reactive oxygen species (ROS) generation, which has regulatory roles in cell morphology and function. The structure of complex I has been determined by X-ray crystallography [24]. It is an L-shaped molecule consisting of an IMM arm that has three or four proton-pumping modules and a matrix arm with iron-sulfur centers that catalyze electron transfer from NADH to the ubiquinol [26]. Complex I is crucial for the normal functioning of most cells, as mutations in its subunits have been linked to a wide range of inherited neuromuscular and metabolic diseases [27]. Rotenone is a bona fide complex I inhibitor that is widely used as a pharmacological tool to dissect the relative contributions of complex I to metabolic alteration and ROS generation. It inhibits electron transfer from the iron-sulfur centers in complex I to the downstream ubiquinol, resulting in electron build-up in the matrix and the formation of ROS [28,29]. 

When faced with mitochondrial inhibition and dysregulated energy production, the cell responds in a multiplicity of ways, with the cell behavior and expression response changing over time. The Electric Cell-substrate Impedance Sensing (ECIS) biosensing technology is uniquely positioned to investigate how the cells dynamically respond to mitochondrial insults, as it monitors the impedance of cells in real-time using a constant 1µA alternating current (AC) [30,31]. The ECIS also is capable of monitoring the spreading activity of cells via capacitance measurements, as well as monitor a cell monolayer’s total electrical resistance. The use of the ECIS then also extends to the investigation of multiple components of the RPE barrier, as the technology uses total resistance measurements to calculate electrical resistance at both the paracellular and basolateral cell surfaces as well as cell membrane capacitance [30,31]. 

In the present study, we aimed to dissect the relative contribution of different mitochondrial bioenergetic parameters to the behavior of RPE cells including spreading, quality of tight junctions, quality of cell–matrix adhesions, and cell membrane morphology and permeability in real-time in live cells using the ECIS biosensor technology. 

## 2. Materials and Methods

### 2.1. Human Retinal Pigmented Epithelial Cell Line (ARPE-19)

ARPE-19 cells were acquired from American Type Culture Collection (ATCC; CRL-2302, Manassas, VA, USA). To culture the cells, Dulbecco’s modified Eagle’s medium-nutrient mixture F-12 (DMEM/F-12, Corning-10-090-CV, Corning, NY, USA) supplemented with 10% Fetal bovine serum (FBS, Corning-35011CV) and 1% penicillin/streptomycin (PS; Hyclone-SV30010, Logan, UT, USA was used. At a time after the ARPE-19 cells achieved confluence on the substrate electrode, they were serum-starved overnight and then treated with the different concentrations of the varied mitochondrial inhibitors in serum-free media (SFM). Treatment was applied once and not removed for the 100 h of monitoring. 

### 2.2. Conducting the ECIS Experiment and Modelling Electric Parameters

The barrier function of ARPE-19 was evaluated over time by regular measurements of the cells’ overall impedance (Z; ohms (Ω)). The device used for this was the Electric Cell-substrate Impedance Sensing (ECIS^®^Zθ (theta)) biosensor technology (Applied Biophysics Inc, Troy, NY, USA, which measured a normalized Z, as previously described [32]. An array of 96-wells (96W20idf PET, Applied Biophysics, Inc.) was coated with 50 µL of 100 µM cysteine for 30 min followed by coating with 50 µL of 0.02% gelatin (Sigma; G1393, Burlington, MA, USA) for 30 min, in order to prepare the electrode substrate array for cultured cells. Thereafter, ARPE-19 cells were seeded in DMEM/F12 full media including 10% FBS and 1% PS at a cell density of 30,000 cells per well. After the ECIS measures the capacitance of the ARPE-19 cells below 20 nF indicating that they have achieved a confluent monolayer on the electrode substrate, the media were changed to SFM for 16–20 h followed by a single continuous treatment with either the control vehicle (DMSO, Sigma), FCCP (Agilent, Santa Clara, CA, USA, 1 and 10 µM), oligomycin (Sigma, 1 and 10 µM), or rotenone (Sigma, 2 and 20 µM). 

The impedance across the ARPE-19 monolayer was measured with respect to both time and to the frequency of the 1µA AC current applied to the substrate electrode. The current frequencies applied were 250, 500, 1000, 2000, 4000, 8000, 16,000, 32,000, and 64,000 Hz. Measurements were taken at an interval of fixed 180-s. Normalized impedance was calculated as the ratio of the raw impedance at each time point to the baseline impedance measured at the last time point before the addition of the treatment. These normalized impedances were then plotted as a function of time. The ECIS software calculates both the resistance and capacitance of the ARPE-19 cell monolayer as a function of this measured total impedance. The ECIS software is also able to resolve the total resistance value into three separate electrical parameters: R_b_ (the paracellular electrical resistance between ARPE-19 cells, ohms-cm^2^), α (the basolateral resistance between the ARPE-19 and its substrate, ohms-cm^1/2^), and C_m_ (the capacitance at the ARPE-19 cell membrane, µF/cm^2^). R_b_ embodies the strength of tight junctions. α represents the degree of attachment of the ARPE-19 cells to its substrate. Since the current model used and the ECIS calculations do not make mathematical distinctions between the capacitance at the basolateral membrane or the apical membrane, the C_m_ calculated is considered to be the same value at both membranes. 

After collection of impedance values and calculation of raw resistances, the resistance for each well was normalized by taking a ratio of the raw resistance at each time point to the baseline resistance measured in that well at the last time point before treatment addition, time T = 0. This normalized resistance was plotted with respect to time. Then the ECIS software modeled the three parameters of the total resistance. In order to compare how the ARPE-19 cells differentially responded to treatment throughout the entire 100 h of experimentation, the area under the curve (AUC) of normalized resistance was chosen as the primary outcome. This measurement allows for a better comparison of whether cells responded differentially throughout the entire treatment interval, instead of at the endpoint or selected middle points. 

### 2.3. Assessment of Varied Mitochondrial Inhibitors on Cell Viability

The effect of different concentrations of the mitochondrial inhibitors FCCP, oligomycin, and rotenone on the viability of ARPE-19 cells was achieved using lactate dehydrogenase (LDH) Cytotoxicity Assay (CyQUANT™; Invitrogen-C20300, Waltham, MA, USA). Briefly, ARPE-19 Cells were grown in 96-well plates (1 × 10^4^/200 μL/well). After incubation with a control vehicle, FCCP (1 or 10 μM), oligomycin (1 or 10 µM), and rotenone (2 or 20 µM) for a specified amount of time (24, 48, or 72 h), the amount of LDH released into the medium was determined spectrophotometrically using a microplate reader (Synergy HI Hybrid Reader, BioTek, Winooski, VT, USA) following manufacturer’s instructions. The 680-nm absorbance value (background signal from the instrument) was subtracted from the 490-nm absorbance value. 

### 2.4. Statistical Analysis

The analytical comparisons of the experimental groups were assessed using the two-tailed *t*-test or one-way analysis of variance (ANOVA) followed by Tukey post-test. Graphical representations of p values are * *p* ≤ 0.05, ** *p* ≤ 0.01, *** *p* ≤ 0.001, **** *p* ≤ 0.0001. The figure legends include the number of biological replicates. 

## 3. Results

### 3.1. Comparative Effects of Different Mitochondrial Components on RPE Barrier Functionality Using Real-Time Bioimpedance Analysis

**Figure 1:**
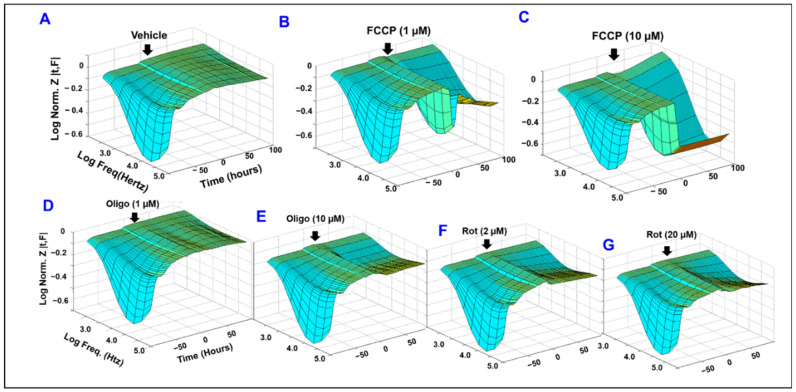
Comparative Effects of different mitochondrial components on RPE barrier functionality using real-time bioimpedance analysis. Effects of FCCP, oligomycin (oligo), and rotenone (Rot) on the barrier function of ARPE-19 cells as measured in real-time by the ECIS system. Three-dimensional plots of Log Normalized Impedance (Z) as a function of both time and of log of the frequency of the 1 µA alternating current (AC) applied to the ECIS electrode. Treatments were applied to the cell groups at T = 0, which was 94 h after cells were placed onto the ECIS electrode, a point at which the cells had already become confluent. Impedance across the cells was measured for 100 h after addition of either control vehicle (**A**), FCCP at concentrations of 1 µM or 10 µM ((**B**,**C**), respectively), oligomycin at concentrations of 1 µM or 10 µM ((**D**,**E**), respectively), or rotenone at concentrations of 2 µM or 20 µM ((**F**,**G**), respectively). Z_0_, the impedance at T_0_, was normalized to a value of 1, and all other impedance values were calculated as the ratio Z_t_/Z_0_. AC current frequencies used were 250, 500, 1000, 2000, 4000, 8000, 16,000, 32,000, and 64,000 Hz. Abbreviations: Z, impedance; Norm, normalized; FCCP, trifluoromethoxy carbonylcyanide phenylhydrazone; Oligo, oligomycin; Rot, rotenone; Freq, frequency; Z_t_, impedance at time t; Z_0_, impedance at time T = 0. Data shown are representative three-dimensional plots of 5–6 independent biological replicates per group.

Given the fact that impaired barrier function of RPE cells is linked to the pathogenesis of AMD and DR, a functional assay using bioimpedance analysis was carried out in vitro to screen the role of different mitochondrial components in maintaining the barrier function of ARPE-19 cells. The specific inhibitors chosen were FCCP (Figure 1B,C) to uncouple ATP synthesis from the accompanying ETC, oligomycin (Figure 1D,E) to inhibit ATP synthase, and Rotenone to inhibit complex I (Figure 1F,G). The treatment with these mitochondrial inhibitors at different concentrations was applied after reaching a plateau in the impedance (Z; y-axis in the 3D model) is reached, which indicates that ARPE-19 cells achieved a confluent monolayer. The barrier integrity was then measured continuously by ECIS technology across the frequency range (250–64,000 Hz; represented on the x-axis in the 3D model) and over a 100-h period (represented as time in hours on the z-axis of the 3D model). From these visual representations of the impedance of ARPE-19 cells as a function of time and as a function of AC frequency (Figure 1A–G), the treatments with the greatest effect on the barrier function of ARPE-19 cells are both FCCP treatments, as those display the most visually dramatic decrease to impedance post-treatment. Less dramatic, though still visualized, losses to impedance are demonstrated in the oligomycin 10 µM and both rotenone treatments. Finally, the oligomycin 1µM treatment at this level appears to demonstrate no impedance differences from the control treatment. These results clearly demonstrate differential roles for mitochondrial components in maintaining RPE cell barrier functionality. 

### 3.2. Real-Time Measurement of Comparative Effects of Specific Mitochondrial Inhibitors on Different Impedance Parameters of RPE Cells

**Figure 2:**
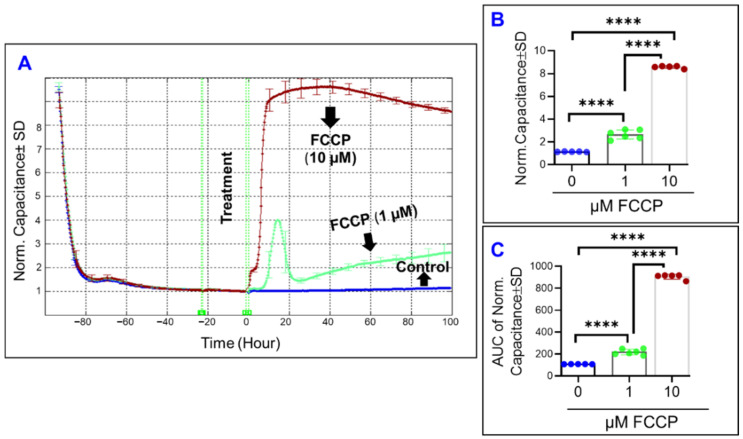
Real-time spreading of ARPE-19 cells in a state of OxPhos uncoupling. (**A**) Normalized capacitance across ARPE-19 cells vs. time, measured at an AC current frequency of 64,000 Hz. Treatments were applied at time T = 0. ARPE-19 cell groups were control, FCCP 1 µM treatment, and FCCP 10 µM treatment. Capacitance was measured from time of placement onto electrode to 100 h after treatment application, and capacitance was normalized to T = 0. (**B**) Bar chart representation of the normalized capacitance of each of the cell groups at time T = 100 h, the experiment’s endpoint. (**C**) Bar chart representation of the area under the normalized capacitance curve of the ARPE-19 cell groups for the interval T = 0–100 h. Abbreviations: AUC, area under the curve; Norm, normalized. Data shown are the mean ± SD of 5–6 independent biological replicates per group. *p* values are **** *p* ≤ 0.0001.

**Figure 3:**
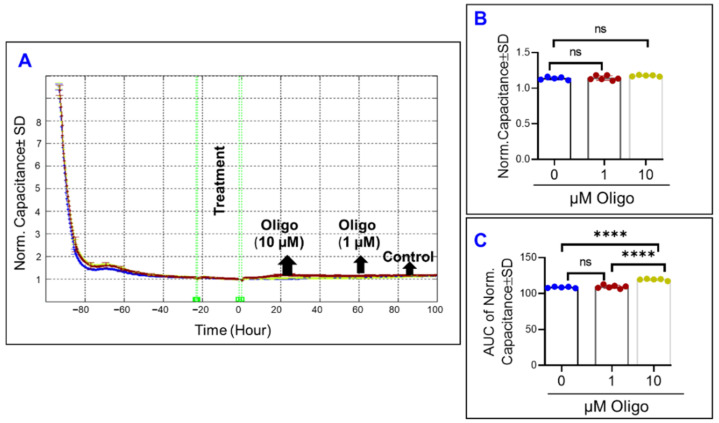
Real-time spreading of ARPE-19 cells under ATP synthase inhibition. (**A**) Normalized capacitance across ARPE-19 cells vs. time, measured at an AC current frequency of 64,000 Hz. Treatments were applied at time T = 0. ARPE-19 cell groups were control, 1 µM, and 10 µM oligomycin treatment. Capacitance was measured from time of placement onto electrode to 100 h after treatment application, and capacitance values were normalized to that at T = 0. (**B**) Bar chart representation of the normalized capacitance of each of the cell groups at time T = 100 h, the experiment’s endpoint. (**C**) Bar chart representation of the area under the normalized capacitance curve of the ARPE-19 cell groups for the interval T = 0–100 h. Compared to the control, the oligomycin 10 µM group affects cell spreading behavior on average across treatment duration, but no effect remains by the end of the experiment. Abbreviations: AUC, area under the curve; Norm, normalized; ns, no significance. Data shown are the mean ± SD of 5–6 independent biological replicates per group. *p* values are **** *p* ≤ 0.0001.

**Figure 4:**
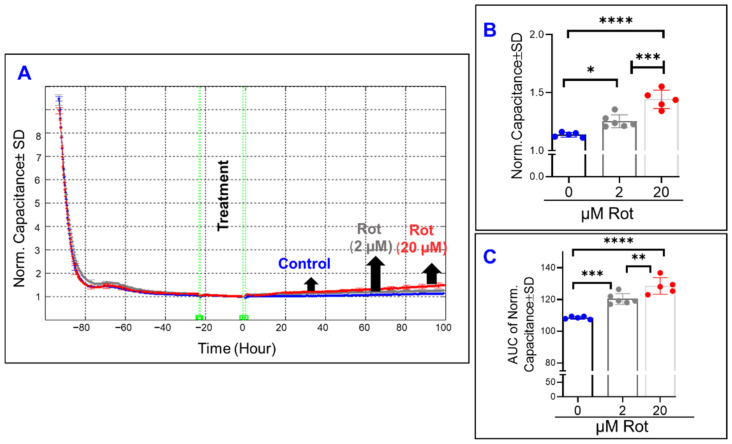
Real-time spreading of ARPE-19 cells under inhibition of complex I of the electron transport chain. (**A**) Normalized capacitance across ARPE-19 cells vs. time, measured at an AC current frequency of 64,000 Hz. Treatments were applied at time T = 0. ARPE-19 cell groups were the control, rotenone 2 µM treatment, and rotenone 20 µM treatment. Capacitance was measured from time of placement onto electrode to 100 h after treatment application, and capacitance values were normalized to that at T = 0. (**B**) Bar chart representation of the normalized capacitance of each of the cell groups at time T = 100 h, the experiment’s endpoint. (**C**) Bar chart representation of the area under the normalized capacitance curve of the ARPE-19 cell groups for the interval T = 0–100 h. Both doses of rotenone disrupted cell spreading during the experiment. Abbreviations: AUC, area under the curve. Data shown are the mean ± SD of 5–6 independent biological replicates per group. *p* values are * *p* ≤ 0.05, ** *p* ≤ 0.01, *** *p* ≤ 0.001, and **** *p* ≤ 0.0001.

Mathematically, the total impedance of cells covering the electrode can be decomposed into two parameters: cell membrane capacitance (C) and cell barrier resistance (R). The former describes how the cells are spreading over the electrode, while the latter includes the resistance at the cell-cell junction, cell-substrate resistance, and cell membrane morphology. Therefore, we aimed to dissect the effect of different mitochondrial components on these two parameters. First, to determine the effect of specific mitochondrial inhibitors on the spreading of ARPE-19 cells over the electrode, the capacitance across the ARPE-19 cells was calculated as a surrogate measurement (Figure 2, Figure 3 and Figure 4). To do so, the frequency at which the maximum ARPE-19 cell’s spreading over the electrode occurs was selected to be 64,000 Hz based on our previously published data [31]. Second, given that the capacitance across the cells decreases in an inverse relationship to the extent of spreading, determining the effects of the mitochondrial inhibitors on the electrical properties of the cells were best performed once the cells had finished spreading across the substrate, and thus had reached a state of electrical quiescence. ARPE-19 cells are considered to have achieved maximum spreading once the capacitance across the cells has reached a stable minimum after placement on the electrode (Figure 2, Figure 3 and Figure 4). Thereafter, the mitochondrial inhibitors were added to the ARPE-19 cells. As shown in Figure 2A, the capacitance of ARPE-19 cells very rapidly accelerates after FCCP (10 µM) treatment, however, the capacitance of ARPE-19 cells takes a matter of a few hours to respond to the lower concentration of FCCP (1 µM). Furthermore, ARPE-19 cells treated with FCCP (1 µM) display a characteristic capacitance that increases from the baseline, reaches a momentary maximum, decelerates for a few hours, and then returns to increase for the remainder of the experiment. This likely represents dynamic changes to the spreading movement of the ARPE-19 cells after 1 µM FCCP treatment. By the end of the experiment (Figure 2B), FCCP caused a dose-dependent increase to the capacitance measured at the experimental endpoint of T = 100 h. To determine if the FCCP effects on capacitance were dose-dependent throughout the entire duration of the experiment, the area under the curve (AUC) was calculated for each of the capacitance curves (Figure 2C). Each FCCP group still demonstrated a significant difference in their AUCs, providing support to the idea that OxPhos uncoupling had a dose-dependent effect on cell capacitance throughout the entire experimental duration. 

In contrast to the effects of OxPhos uncoupling, inhibition of ATP synthase by oligomycin had a much less demonstrable effect on ARPE-19 capacitance (Figure 3A). Both oligomycin treatments had no effect on the final ARPE-19 capacitance at the end of the experiment (Figure 3B). In addition, when the AUCs are compared in Figure 3C, only oligomycin at 10 µM causes a small (~10%) increase in capacitance throughout the entire experiment, yet 1 µM of oligomycin had no effect compared to control. The capacitance of the oligomycin (10 µM) treated cells differs throughout the experiment but not at the end, indicating that the effect of the ATP synthase inhibition on the spreading of ARPE-19 is reversible. 

On the other hand, inhibiting complex I of the mitochondrial ETC by rotenone changed the spreading of ARPE-19 across the electrode throughout the experiment (Figure 4A). While the capacitance curves (Figure 4A) only display small changes off the baseline for each of the rotenone groups, the endpoint capacitance comparison (Figure 4B) and the AUC capacitance comparison (Figure 4C) both show how rotenone causes a dose-dependent increase in capacitance both at the end and throughout the experiment. These results indicate that the effect of Complex I inhibition on the spreading of ARPE-19 cells is irreversible, as mid-experiment changes were carried to the experiment’s end. 

### 3.3. Real-Time Monitoring of the Comparative Effects of Different Mitochondrial Components on the Total Resistance of ARPE-19 Cells

**Figure 5:**
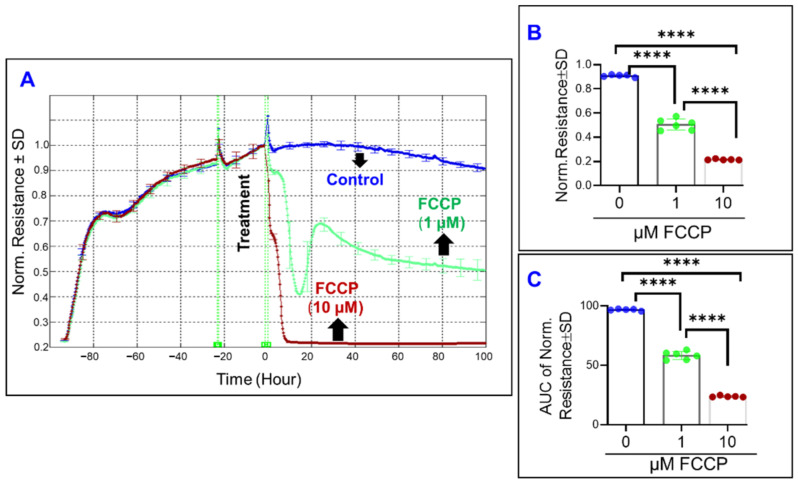
Real-time measurement of the total resistance across the ARPE-19 cells in a state of OxPhos uncoupling. (**A**) Plot of normalized resistance vs. time for the cell groups of the control, FCCP 1 µM, and FCCP 10 µM. Resistance was measured from the time the cells were placed on the ECIS electrode to the time 100 h after treatment application. Resistance was normalized to T = 0, the time of treatment application. (**B**) Bar chart representation of each group’s normalized resistance at the endpoint of the experiment, T = 100 h. Comparison between all three groups displays a significant difference. (**C**) Bar chart representation of the areas under the normalized resistance curve for the interval T = 0–100 h. Using AUC, all three groups demonstrate a significant difference from each other. Abbreviations: AUC, area under the curve. Data shown are the mean ± SD of 5–6 independent biological replicates per group. *p* values are **** *p* ≤ 0.0001.

**Figure 6:**
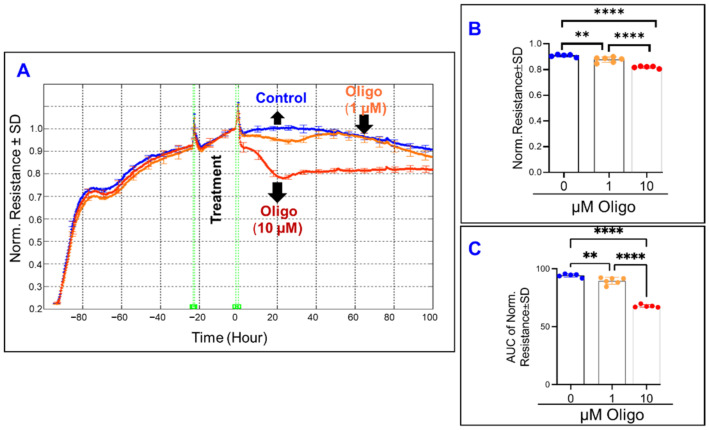
Real-time measurement of the total resistance across the ARPE-19 cells in a state of inhibited ATP synthase. (**A**) Plot of normalized resistance vs. time for the cell groups of the control, 1 µM, and 10 µM Oligomycin (Oligo). Resistance was normalized to T = 0, the time of treatment application. (**B**) Bar chart representation of each group’s normalized resistance at the endpoint of the experiment, T = 100 h. Comparison between all three groups displays a significant difference. (**C**) Bar chart representation of the areas under the normalized resistance curve for the interval T = 0–100 h. Using AUC, all three groups demonstrate a significant difference from each other. Abbreviations: Norm, normalized; AUC, area under the curve. Data shown are the mean ± SD of 5–6 independent biological replicates per group. *p* values are ** *p* ≤ 0.01 and **** *p* ≤ 0.0001.

**Figure 7:**
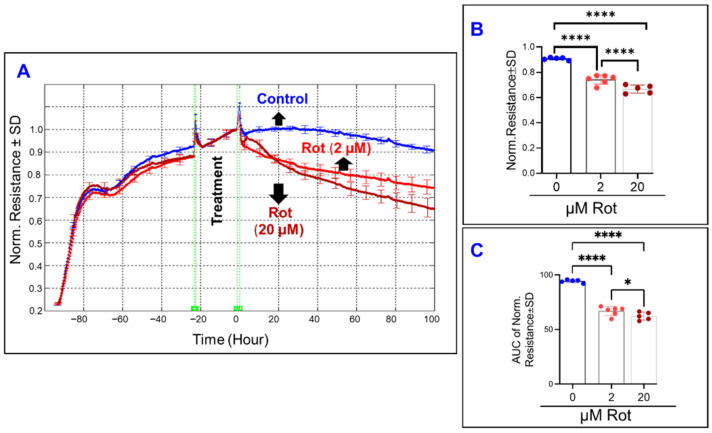
Real-time measurement of the total resistance across the ARPE-19 cells in a state of ETC complex I inhibition. (**A**) Plot of normalized resistance vs. time for the cell groups of the control, 2 µM and 20 µM rotenone (Rot). Resistance was measured from the time the cells were placed on the ECIS electrode to the time 100 h after treatment application. Resistance was normalized to T = 0, the time of treatment application. (**B**) Bar chart representation of each group’s normalized resistance at the endpoint of the experiment, T = 100 h. Comparison between all three groups displays a significant difference. (**C**) Bar chart representation of the areas under the curve (AUC) of normalized resistance for the interval T = 0–100 h. Using AUC, all three groups demonstrate a significant difference from each other, and this AUC difference between the groups demonstrates a smaller *p*-value of difference between the rotenone group compared to that found in the endpoint comparison in B. Abbreviations: Norm, normalized; AUC, area under the curve. Data shown are the mean ± SD of 5–6 independent biological replicates per group. *p* values are * *p* ≤ 0.05 and **** *p* ≤ 0.0001.

Next, we sought to determine the effect of specific mitochondrial inhibitors on the overall barrier functionality of ARPE-19 cells by measuring total electrical resistance across the cells. To achieve this goal, the frequency where the total resistance is at a maximum was determined to be 4000 Hz, as in our previously reported study [31]. Thereafter, the ARPE-19 cell groups’ total resistances were measured for 100 h after treatment with different mitochondrial inhibitors (Figure 5, Figure 6 and Figure 7). In Figure 5A the effect of FCCP, and thus OxPhos uncoupling, on total resistance as a function of time is depicted. FCCP causes a dose-dependent reduction in cell resistance both at the endpoint of the experiment (Figure 5B) and throughout the entire experiment (Figure 5C). As such, it can be inferred that uncoupling the ETC from ATP synthesis has a significantly detrimental and irreversible effect on the RPE barrier function. 

In a pattern similar to the effect of FCCP, oligomycin-induced ATP synthase inhibition (Figure 6A) caused a dose-dependent decrease to both the endpoint resistance (Figure 6B) and the total resistance throughout the experiment (Figure 6C). Then in Figure 7A, the effect of inhibiting complex I of the mitochondrial ETC is seen. Increasing the concentration of rotenone causes further decreases to the total resistance measured at the end of the experiment (Figure 7B), and the same is seen when the AUC of resistance is compared between the rotenone groups and the control (Figure 7C). However, when comparing Figure 7B,C and appreciating the difference between the two rotenone groups in both, we see that the strength of the difference is stronger when comparing their endpoint resistances as opposed to comparing their resistances throughout the experiment, suggesting a possible time dependence of the effect of complex I inhibition on total resistance. 

### 3.4. Comparative Effects of Different Mitochondrial Inhibitors on the Components of ARPE-19 Cells’ Total Resistance

**Figure 8:**
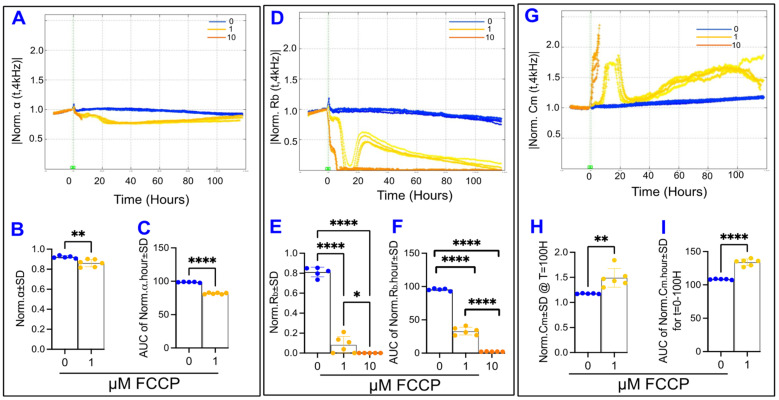
Real-time measurement of α, R_b_, and C_m_ in ARPE-19 cells in a state of OxPhos uncoupling. (**A**) Normalized α measured at 4000 Hz vs. time (T) for the interval from the start of treatment at T = 0–100 h after initiation of treatment with an FCCP concentration of 0, 1, or 10 µM. The α curves for the FCCP 10 µM cell group all abruptly end around the 5.1-h mark, as this is the time when the R_b_ curves for the FCCP 10 µM group reach zero. The ECIS software is unable to calculate a positive integer for α at times when R_b_ has a value of zero. By this 5.1-h mark, the endpoint α values and the area under the curve (AUC) of α are significantly different between each FCCP group and control. These data are not shown in the figure. (**B**) End-point values for the control and FCCP 1 µM groups normalized α at the end of the experiment, where FCCP 1 µM has decreased from the control. (**C**) AUC of normalized α for the interval T = 0–100 h for the groups FCCP 0 (Control) and 1 µM. Throughout the total experiment, the FCCP 1 µM group demonstrates a difference in behavior from the control group. (**D**) Normalized R_b_ measured at 4000 Hz vs. time (T) for the interval from the addition of treatment at T = 0 to 100 h afterward. The R_b_ curves for the FCCP 10 µM cell group reach zero around the 5.1 h mark. (**E**) R_b_ experiment endpoint comparisons for all three groups, where a dose-dependent effect on end R_b_ is seen. (**F**) AUC of normalized R_b_ for the time from treatment start to experiment end. FCCP treatment demonstrates a dose-dependent reduction in R_b_. (**G**) Normalized C_m_ measured at 4000 Hz vs. time (T) for the interval from the start of treatment at T = 0 to 100 h after initiation of treatment with an FCCP concentration of 0, 1, or 10 µM. The C_m_ curves for the FCCP 10 µM cell group all abruptly end around the 5.1 h mark, as this is the time when the R_b_ curves for the FCCP 10 µM group reach zero. The ECIS software is unable to calculate a positive integer for C_m_ at times when R_b_ has a value of zero. At this 5.1 h mark, the endpoint C_m_ values and the area under the curve (AUC) of C_m_ are significantly different between FCCP 10 µM and FCCP 0 but not between FCCP 10 µM and Control. These data are not shown in the figure. (**H**) Comparison of C_m_ at the experimental endpoint of T = 100 h for the control and FCCP 1 µM groups, where FCCP 1 µM has resulted in an endpoint difference from FCCP 0. (**I**) AUC of normalized C_m_ for the interval T = 0–100 h for the groups FCCP 0 and 1 µM. Throughout the experiment, the FCCP 1 µM group demonstrates a difference in C_m_ behavior from the control group. Statistical comparison analysis in (**B**,**C**,**H**,**I**) done using two-tailed unpaired *t*-tests. Statistical comparison analysis in (**E**,**F**) done using the ANOVA test followed by Tukey post hoc test to make the comparisons. Abbreviations: Norm, normalized; AUC, area under the curve; ns, no significance. Data shown are the mean ± SD of 5–6 independent biological replicates per group. *p* values are * *p* ≤ 0.05, ** *p* ≤ 0.01, and **** *p* ≤ 0.0001.

The ECIS system has the capability of deconvoluting the total resistance measured across the cells into three distinct components: R_b_, the paracellular resistance component; α, the basolateral resistance component; and C_m_, the capacitance at the cell membrane. These parameters are calculated by fitting a mathematical model developed by Giaever and Keese [33]. Figure 8 demonstrates the effect of FCCP on each of the components of total resistance. First, in Figure 8A, the α curve for FCCP 10 µM abruptly ends at T = 5.1 h, which is the time when the R_b_ curve for that group reaches 0, as seen in Figure 8D. The ECIS is only able to calculate a real value for α at times when R_b_ is a non-zero positive value. Both the FCCP groups displayed a change to α within the first 5.1 h after treatment, before the FCCP 10 µM curve ends, where FCCP causes reductions in α in a dose-dependent manner (data not shown). Then, when regarding the entire 100 h of the experiment, the FCCP 1 µM treatment resulted in not only a reduction in α from control at the endpoint (Figure 8B) but also reduced α throughout the whole experiment, represented by the AUC in Figure 8C. Second, Figure 8D displays the effect of OxPhos uncoupling on normalized R_b_ over time. FCCP causes a dose-dependent reduction in both the endpoint R_b_ values and the behavior of R_b_ throughout the experiment (Figure 8E,F, respectively). In fact, FCCP 10 µM treatment completely eliminates the resistance contribution of R_b_, as this group’s R_b_ reaches and maintains zero around T = 5.1 h. In addition, the FCCP 1µM group displays an interesting trend where it approaches and reaches zero around 10 h, increases dramatically from about hour 15 to hour 25, and then descends gradually back to zero by hour 100. Next, Figure 8G shows C_m_ over time after treatment with FCCP, and again we see that the curve related to FCCP 10 µM ends abruptly at 5.1 h because the ECIS cannot calculate real values for C_m_ when R_b_ is zero. The capacitance of the cell membrane in the FCCP 10 µM group is significantly increased both at the end of the 5.1 h and throughout them, whereas no changes have occurred to the FCCP 1 µM group yet (data not shown). When considering the C_m_ across the entire experiment, FCCP 1 µM treatment results in a final C_m_ that is elevated over the control (Figure 8H) and elevates this capacitance at the cell membrane throughout the experiment interval (Figure 8I). Taken together, the results seen in Figure 8 indicate that FCCP, and thus OxPhos uncoupling, disrupts all three components of the total resistance and the barrier of the ARPE-19 cells. 

**Figure 9:**
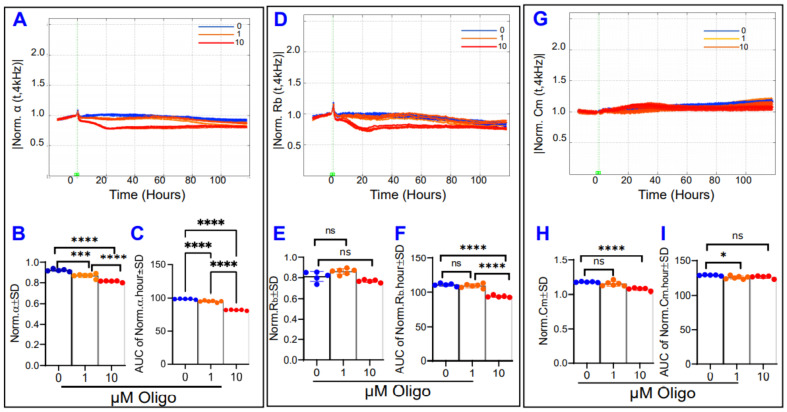
Real-time measurement of α, R_b_, and C_m_ in ARPE-19 cells in a state of ATP synthase inhibition. (**A**) Normalized α measured at 4000 Hz vs. time (T) from the start of treatment at T = 0 to 100 h after initiation of treatment with an oligomycin concentration of 0, 1, or 10 µM. (**B**) α values at the experimental end T = 100 h, both oligomycin (Oligo) groups demonstrate differences from control (Oligo 0). (**C**) Areas under the curve (AUC) of normalized α for the time from treatment start to experiment end. By the end of the experiment, ATP synthase inhibition has resulted in significant reductions to α in a dose-dependent fashion. (**D**) Normalized R_b_ measured at 4000 Hz vs. time (T) for the interval from the start of treatment at T = 0 to 100 h after initiation of treatment. R_b_ values at T = 100 h, where the 10 µM but not the 1 µM Oligo group had an effect. (**E**) R_b_ values at the experimental end T = 100 h, both Oligo groups demonstrate no differences from control. (**F**) AUC of normalized R_b_ for the time T = 0–100 h. Oligomycin treatment results in a reduction in R_b_ at high doses, and there is no significant change to R_b_ at low doses. (**G**) Normalized C_m_ measured at 4000 Hz vs. time (T). (**H**) C_m_ values measured at T = 100 h, where only the high dose oligomycin had an effect on endpoint C_m_. (**I**) AUC of normalized C_m_ for treatment interval. The behavior of the oligomycin 1 µM group throughout the entire experiment time was slightly different from control (Oligo 0), while the behavior of the 10 µM Oligo group did not differ over this time frame. Abbreviations: Norm, normalized; AUC, area under the curve; ns, no significance. Data shown are the mean ± SD of 5–6 independent biological replicates per group. *p* values are * *p* ≤ 0.05, *** *p* ≤ 0.001, and **** *p* ≤ 0.0001.

Next, Figure 9 now displays the barrier components over time after treatment with oligomycin. High-dose oligomycin (10 µM) causes both α (Figure 9A–C) and R_b_ (Figure 9D–F) to significantly decrease, both at the end and throughout the duration of the experiment. However, low-dose oligomycin (1 µM) also had a strong reductive effect on α but had no effect on R_b_ at the endpoint or throughout the experimental period. In a curious result, the effect of ATP synthase inhibition on C_m_ (Figure 9G) was a reduction of C_m_ at the end of the experiment for only the oligomycin 10 µM group (Figure 9H), yet only the oligomycin 1 µM group differed from controls significantly throughout the experiment (Figure 9I). Collectively, the results in Figure 9 indicate that ATP synthase inhibition by oligomycin only significantly affects the basolateral component of the RPE barrier, suggesting ATP is a necessary factor in maintaining a strong cell-substrate attachment. 

**Figure 10:**
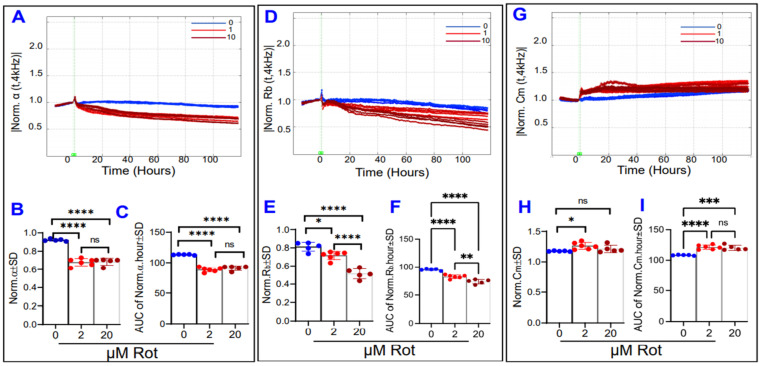
Real-time measurement of α, R_b_, and C_m_ in ARPE-19 cells in a state of ETC complex I inhibition. (**A**) Normalized α measured at 4000 Hz vs. time (T) from the start of treatment at T = 0 to 100 h after initiation of treatment with a rotenone (Rot) concentration of 0, 2, or 20 µM. (**B**) Comparison of α values measured at T = 100 h, the end of the experiment. Both low and high-dose Rot had the same effect on α endpoints. (**C**) Areas under the curve (AUC) of normalized α for the time from treatment start to experiment end. By the end of the experiment, complex I inhibition resulted in significant reductions to α in a non-dose-dependent fashion. (**D**) Normalized R_b_ measured at 4000 Hz vs. time (T) for the interval T = 0–100 h. (**E**) Comparison of R_b_ values at T = 100 h. Rot exerted a dose-dependent effect on endpoint R_b_. (**F**) Areas under the curve of normalized R_b_ for the time T = 0–100 h. Rot treatment also had a dose-dependent effect on the R_b_ behavior throughout the experiment. (**G**) Normalized C_m_ measured at 4000 Hz vs. time (T) from T = 0–100 h. (**H**) Comparison of C_m_ values measured at the experimental end, where only low dose changed the C_m_. (**I**) Areas under the curve of normalized C_m_ for the time T = 0–100 h. Both Rot treatments resulted in C_m_ alterations compared to control within the 100 h after treatment, though these Rot groups did not differ from each other. Abbreviations: Norm, normalized; AUC, area under the curve; ns, no significance. Data shown are the mean ± SD of 5–6 independent biological replicates per group. *p* values are * *p* ≤ 0.05, ** *p* ≤ 0.01, *** *p* ≤ 0.001, and **** *p* ≤ 0.0001.

Lastly, Figure 10 examines the effects of rotenone on the components of the electrical barrier. Both high and low dose rotenone reduced α with the same intensity by the endpoint and throughout the experiment (Figure 10A–C), whereas rotenone’s reductive effect on R_b_ was dose-dependent both at the endpoint and throughout (Figure 10D–F). Rotenone at a low dose was associated with an increase in C_m_ at the experiment’s end (Figure 10G–H), whereas both low and high dose rotenone had altered C_m_ throughout the experiment (Figure 10I). Taken as a whole, it appears that complex I inhibition mostly affects the ARPE-19 paracellular resistance R_b_ compared to basolateral resistance α or membrane capacitance C_m_ as this was the only parameter of the three to respond in a dose-dependent fashion. 

### 3.5. Comparative Effects of Different Mitochondrial Inhibitors on ARPE-19 Cell Viability

**Figure 11:**
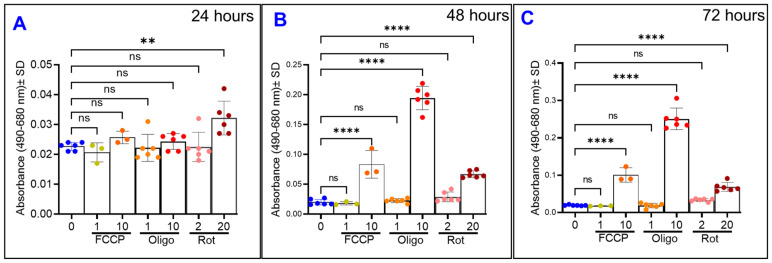
Effects of mitochondrial inhibitors on ARPE-19 cell viability. Lactate dehydrogenase (LDH) cytotoxicity assays were done at T = 24, 48, and 72 h after initiation of treatment. (**A**) LDH release of the cell groups 24 h after experiment start. Only rotenone 20 µM is associated with increased LDH release. (**B**) LDH release at T = 48 h. At this time, the FCCP 10 µM and oligomycin 10 µM have joined the rotenone 20 µM with associated increased LDH release. (**C**) LDH release of the cell groups at 72 h. Abbreviations: FCCP, trifluoromethoxy carbonylcyanide phenylhydrazone; Oligo, oligomycin; Rot, rotenone; ns, no significance. Data shown are the mean ± SD of 5–6 independent biological replicates per group. *p* values are ** *p* ≤ 0.01 and **** *p* ≤ 0.0001.

**Figure 12:**
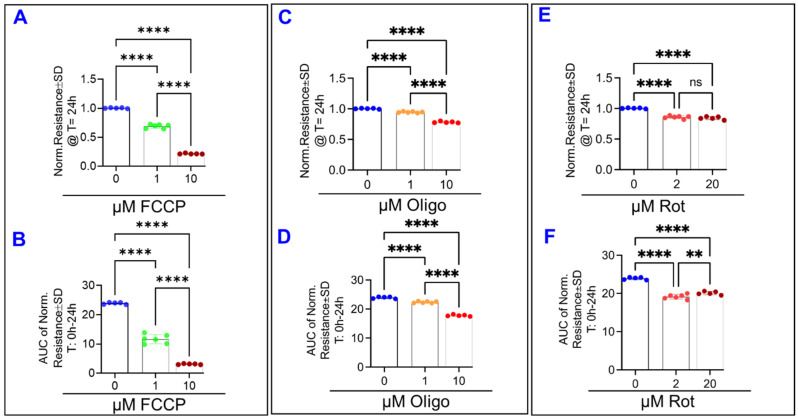
Effects of mitochondrial inhibitors on ARPE-19 cells total resistance at experimental midpoint T = 24 h. (**A**) Midpoint resistance at T = 24 h for the ARPE-19 cells treated with FCCP. After 24 h of treatment with FCCP, there is a dose-dependent effect of FCCP on the midpoint resistance. (**B**) Area under the curve (AUC) of total resistance for the cells treated with FCCP, on the interval T = 0–24 h. Like the 24 h midpoint resistance for the FCCP-treated cells, there is a dose-dependent effect. (**C**) Midpoint resistance at T = 24 h for the ARPE-19 cells-treated with oligomycin. After 24 h of treatment with oligomycin, there is a dose-dependent decrease in the resistance. (**D**) Area under the curve (AUC) of total resistance for the cells treated with oligomycin, on the interval T = 0–24 h. The AUC comparison shows the same dose-dependent effect of oligomycin that the endpoint comparison does. (**E**) Midpoint resistance at T = 24 h for the ARPE-19 cells treated with rotenone. After 24 h of treatment with rotenone, both doses have reduced resistance to the same level. (**F**) Area under the curve (AUC) of total resistance for the cells treated with rotenone, on the interval T = 0–24 h. Abbreviations: Norm, normalized; FCCP, trifluoromethoxy carbonylcyanide phenylhydrazone; Oligo, oligomycin; Rot, rotenone; ns, no significance. Data shown are the mean ± SD of 5–6 independent biological replicates per group. *p* values are ** *p* ≤ 0.01 and **** *p* ≤ 0.0001.

Cytotoxicity assays of all the ARPE-19 cell groups were performed at 3 distinct time points during the experiment by measuring the release of lactate dehydrogenase (LDH) (Figure 11). Time points used were 24, 48, and 72 h post-treatment. At the 24 h mark, as shown in Figure 11A, only the rotenone 20 µM treatment has caused increased LDH release. This contrasts to Figure 5A, Figure 6A and Figure 7A, where clear reductions to total resistance have already occurred in both FCCP groups (Figure 5A), both oligomycin groups (Figure 6A), and both rotenone groups (Figure 7A) by this 24-h mark. Especially notable is how the resistance of the FCCP 10 µM group has plateaued to a minimum for several hours before this time point, yet Figure 11A shows this group has not lost viability yet. After 48 h, as shown in Figure 11B, the higher doses of the three types of mitochondrial inhibitors have each caused LDH release. Finally, at the 72-h mark as displayed in Figure 11C, changes to viability are still only seen in the FCCP 10 µM, oligomycin 10 µM, and rotenone 20 µM groups. To compare this timing of cell viability’s loss to resistance changes, the 24 h midpoint resistances for the groups were analyzed (Figure 12). After 24 h of treatment with FCCP, a dose-dependent decrease in total resistance is already observed (Figure 12A,B). Comparing this to the LDH release of the FCCP treated cells in Figure 11A, both the FCCP 1 µM and 10 µM treated cells demonstrated loss to resistance before their viability was compromised. The same can be said of the oligomycin treated cells, as after 24 h oligomycin exhibited a dose-dependent decrease in resistance (Figure 12C,D), whereas Figure 11A demonstrates no changes to these cells’ viability. Finally, when analyzing the changes to resistance in the rotenone-treated cells, both rotenone 2 µM and 20 µM resulted in the resistance losses after 24 h (Figure 12E,F). This contrasts to Figure 11A where only rotenone 20 µM has affected viability, and again shows how the ECIS-measured resistance losses in the rotenone 2 µM cells before viability was altered. Altogether, these results show how real-time measurement of the ARPE-19 resistance by the ECIS can pick up on changes to cell barrier functionality before cells’ lost viability is measured. 

## 4. Discussion

The key finding of our study is that mitochondrial OxPhos components contribute differentially to maintaining ARPE-19 cell functionality. More specifically, the uncoupling of OxPhos disrupts the barrier integrity of ARPE-19 cells across three distinct domains: the barrier between cells (R_b_), the attachment between cells, and their basolateral substrate (α), and the barrier to flow through the cell membrane (C_m_). On the other hand, ATP synthase inhibition mostly affects the ARPE-19 cells’ attachment to their substrate (α), which is contrary to complex I inhibition, which mostly affects the ARPE-19 paracellular resistance R_b_. The following evidence supports this conclusion. (a) FCCP and thus OxPhos uncoupling disrupts the barrier function in the ARPE-19 cells at all 3 components of the total resistance (Rb, α, and C_m_) in a dose-dependent manner, (b) oligomycin and thus ATP synthase inhibition caused a significant decrease in α resistance in a dose-dependent manner, (c) rotenone’s complex I inhibition had the greatest effect on the paracellular component of the RPE barrier, as R_b_, but not α or C_m_, was affected in a dose-dependent fashion here; and (d) interestingly, this breakdown effect of different mitochondrial inhibitors on the barrier function of ARPE-19 cells was not a consequence of RPE cell death. Our study is the first to show these temporal relationships between RPE barrier parameters in response to various OxPhos components inhibitors using ECIS mathematical modeling system. 

Of the three components of total resistance, R_b_ was the one most noticeably affected in the experiment, as treatment with FCCP drove R_b_ close to near zero at both concentrations. The paracellular resistance was also the most vulnerable target for complex I inhibition since rotenone affected the R_b_ component of the RPE barrier in a dose-dependent manner. As total resistance was soundly reduced in each of these treatments, this suggests that a decrease in overall RPE barrier integrity in poor mitochondrial functioning is strongly related to disruption of intercellular attachments, likely either due to reorganization to or direct damage at the tight junctions. A mechanism to explain the breakdown effect on the paracellular barrier common to both ETC uncoupling and complex I inhibition is the accumulation of reactive oxygen species (ROS) inside the mitochondrial matrix, resulting in the release of ROS burst and then losses of adherent junction proteins. FCCP and rotenone have both been shown to increase the production of ROS. FCCP depolarization of mitochondria generates oxygen free radicals in human endothelial cells as well as human pulmonary adenocarcinoma cells [34,35]. Rotenone interrupts electron transfer from the iron-sulfur centers in complex I to the downstream ubiquinol, resulting in electron leakage inside the matrix and the buildup of ROS. At higher ROS levels, evidence suggests that ROS may trigger mitochondrial permeability transition pore (mPTP) induction, which plays an important role in releasing a ROS burst [36] that may destroy adherent junction proteins (AJP). Although AJPs do not constitute a physical barrier to the paracellular diffusion of macromolecules, they are known to indirectly affect the integrity of tight junctions [37]. E-cadherin and β-catenin are the main AJPs localized just beneath the key paracellular junctional protein, zonula occludens (ZO-1) that are affected by ROS generation [38]. ROS has been shown to redistribute E-cadherin and β-catenin into the intracellular compartment from the intercellular junctions and to dissociate E-cadherin and β-catenin interaction [39]. Intercellular attachment losses with disruption of ZO-1, N-cadherin, and β-catenin have even been demonstrated specifically in RPE cells exposed to high ROS levels [40,41]. Therefore, one mechanism by which uncoupling or complex I inhibition disrupts the paracellular barrier function of ARPE-19 cells may involve the ROS-induced AJPs’ damage. 

One intriguing result from the treatment of ARPE-19 with FCCP 1 µM is the undulating behavior to the total resistance and R_b_ at mid-experiment before the trend returns to a steady descent (Figure 5A and Figure 8F). Endogenous uncoupling protein UCP-2 has demonstrated a protective effect against ROS accumulation in ARPE-19 cells [42], so perhaps this temporary recovery to the ARPE-19 barrier is a partial protective effect that uncoupling has against ROS, occurring before the cell is overwhelmed and barriers return to breakdown. 

R_b_ losses due to FCCP treatment may relate to reduced activity of the plasma membrane Na,K-ATPase, from ATP deficiency secondary to mitochondrial uncoupling. Rajasekaran et al. found that inhibition of the Na,K-ATPase decreased transepithelial resistance and increased permeability of human RPE cells [43], which provides a connection between mitochondrial ATP production and RPE paracellular barrier functionality. However, the results from the oligomycin treated ARPE-19 cells in our study did not demonstrate a dose-dependent decrease on R_b_, thus ATP deficiency may not account for all changes to the ARPE-19 paracellular barrier. 

An additional explanation for rotenone-related disruption of intracellular connections is that rotenone treatment of ARPE-19 cells has been shown to lead to mitochondrial DNA damage and mitotic catastrophe with increased autophagy activity [44]. In another of the body’s tightly controlled blood barrier systems, the blood-brain barrier, induced autophagy of in vitro barrier endothelial cells resulted in disruption of cell’s tight junctions [45], including downregulation of the tight junction proteins ZO-1, occludin, and claudin-5. Autophagy of RPE cells under chronic oxidative stress has been shown to be protective against RPE cell death in DR and AMD [46,47], however, dysregulated autophagy in both DR and AMD has been associated with each of their pathogeneses [47,48,49,50]. Taken together, oxidative stress with aberrant autophagy may be important contributions to loss of RPE tight junctions and barrier function. 

Mitochondrial dysfunction also weakened the strength of ARPE-19 cell attachment to its substrate, with FCCP’s ETC-uncoupling and oligomycin’s ATP synthase inhibition each causing a significant and dose-dependent decrease in α, the basolateral resistance. The adhesion of the RPE cell to its substrate is generally controlled by surface integrins ligated with extracellular matrix (ECM) proteins [51,52]. New evidence shows how the essential adhesion component, integrin-linked kinase (ILK), is dynamically involved in this process, and ATP was shown to be an obligatory binding partner for ILK to retain focal adhesion stabilization [53]. ATP’s importance to RPE cell adhesion may also be related to the proper function of the Na,K-ATPase. In a study using MDCK epithelial cells, inhibition of the Na,K-ATPase with ouabain resulted in detachment of cells from both neighboring cells and the substrate [54]. Furthermore, additional studies are needed to test directly for a dissociation between ATP derived from glycolysis versus mitochondria in maintaining RPE cell adhesion to ECM versus maintaining a paracellular barrier to determine which one is used preferentially in each process. 

Mechanisms to explain the FCCP induced changes in C_m_, and thus changes to the ARPE-19 transcellular barrier, are potentially related to changes in ion conductance and permeability at the cell membrane. While one known factor of C_m_ in RPE is its direct relationship to the surface area [55], C_m_ has also been shown to be directly related to ion channel expression and increased membrane permittivity even when the surface area has been artificially reduced [56]. As this study is interested in changes to the RPE barrier, membrane permittivity-related changes to C_m_ represent a prospective barrier alteration. FCCP has demonstrated an ability to acidify cytoplasm [54,57], and the activity of RPE inwardly rectifying K+ channels is known to be altered by decreased intracellular pH, displaying transient activation followed by inhibition [58]. Sodium and hydrogen conductance also may be altered, as FCCP has been shown to increase their currents across the plasma membrane in bovine aortic endothelial cells [59]. 

In summary, our results reveal differential roles for three different OxPhos components in maintaining RPE cell functionality in which the uncoupling of ETC from ATP synthesis is the major factor disrupting the barrier integrity of ARPE-19 cells. Such differences could be used as a screening tool for selective agents that improve the OxPhos coupling efficiency in therapeutic approaches for the treatment of RPE-related retinal diseases. 

## Data Availability

Data associated with this manuscript are available upon request from corresponding author.
